# Biodiversity and distribution of gelatinous macrozooplankton in the North Sea and adjacent waters - dataset from winter 2022

**DOI:** 10.1016/j.dib.2024.111100

**Published:** 2024-11-02

**Authors:** Camilla Juul Dahl Jensen, Louise G. Køhler, Bastian Huwer, Malin Werner, Leslie Cieters, Cornelia Jaspers

**Affiliations:** a Centre for Gelatinous Zooplankton Ecology and Evolution, National Institute of Aquatic Resources, Technical University of Denmark, Kemitorvet 202, 2800 Kgs. Lyngby, Denmark; b National Institute of Aquatic Resources, Technical University of Denmark, Kemitorvet 201, 2800 Kgs. Lyngby, Denmark; c Institute of Marine Research, Department of Aquatic Resources (SLU Aqua), Swedish University of Agricultural Sciences, Turistgatan 5, S-453 30 Lysekil, Sweden

**Keywords:** Biodiversity, Jellyfish, Comb jelly, Midwater ring net survey (MIK), Good Environmental Status (GES), Marine Strategy Framework Directive (MSFD), International Bottom Trawl Survey (IBTS)

## Abstract

The diversity and distribution of gelatinous macrozooplankton is described by presenting qualitative and quantitative data of the jellyfish and comb jelly community encountered in the North Sea and Skagerrak/Kattegat during January/February 2022. Data were generated as part of the North Sea Midwater Ring Net (MIK) survey [1], an ichthyoplankton survey conducted at night-time during the quarter 1 (Q1) International Bottom Trawl Survey (IBTS), aboard the Danish R/V DANA (DTU Aqua) and the Swedish R/V Svea (SLU). A total of 100 stations were investigated using a 13 m long Midwater Ring Net (MIK net) with an opening diameter of 2 m and a mesh size of 1.6 mm, which is 0.5 mm meshed for the last meter of the net and the cod end [2]. Samples were collected by double oblique hauls from the surface to 5 m above the seafloor [2]. Twelve gelatinous macrozooplankton species were encountered during the Q1 2022 survey. Species encountered included the hydrozoan jellyfish i) *Aequorea vitrina*, ii) *Aglantha digitale*, iii) *Clytia* spp., iv) *Leuckartiara octona*, v) *Tima bairdii*, vi) *Muggiaea atlantica*; the two scyphozoan jellyfish i) *Cyanea capillata* and ii) *Cyanea lamarckii* as well as the comb jelly (ctenophora) species i) *Beroe* spp., ii) *Bolinopsis infundibulum*, iii) *Pleurobrachia pileus* and iv) the non-indigenous *Mnemiopsis leidyi*. In total 4882 individual specimens from samples and sub-samples were analyzed and extrapolated to 71,888 records of gelatinous macrozooplankton in the investigation area. For rare species, the entire sample was analyzed, while for abundant taxa, sub-samples were used to assess abundances. The raw counts were converted to volume-specific densities (individuals m^-3^) and area-specific abundances (individuals m^-2^), based on calibrated flow meter recordings and recorded maximum depth of the MIK net during each haul. Further, size data for the different species were obtained from a total of 4775 individual gelatinous macrozooplankton organisms. Size data are presented in the accompanying database and was used to calculate species-specific wet weights, using published size-weight regressions [3]. In addition, we present spatial distribution patterns of the weight specific biomass for the total gelatinous macrozooplankton community as well as the sub-groups i) hydrozoa, ii) scyphozoa and iii) ctenophora across the investigation area. The presented data contribute to a baseline describing the gelatinous macrozooplankton diversity and distribution in the extended North Sea area during winter [3,4] and summer [5]. The data can contribute to address the question if gelatinous macrozooplankton densities increase due to global change pressures and will help to understand their interaction with commercially important fish species, which are assessed during the same surveys. As such, this data paper presents a valuable resource on biodiversity and non-indigenous species records and highlights the importance of monitoring gelatinous macrozooplankton to facilitate an ecosystem approach to assess if the ecosystem state meets a ‘good environmental status (GES)’, as demanded by the EU Marine Strategy Framework Directive (MSFD).

Specifications Table

**Table:** 

Subject	Biodiversity Marine Biology Systematics, Ecology and Behavior Oceanography
Specific subject area	Spatial distribution of the gelatinous macrozooplankton community in the North Sea/Skagerrak/Kattegat during winter 2022. Species-specific densities, size-distributions and wet weights are provided for 15 species including Hydrozoans, Scyphozoans and Ctenophora.
Type of data	2 Tables 9 Figures 2 Appendices (summary and raw data tables)
Data collection	Quantitative gelatinous macrozooplankton data were collected at 100 stations in the extended North Sea area, Northern Europe during the Midwater Ring Net (MIK) survey, as part of the International Bottom Trawl Survey (IBTS). During night-time, a 13 m long Midwater Ring Net (MIK net, 2 m diameter, mesh size 1.6 mm, mesh size in last 1 m of net & in cod end: 500 µm) was used to collect gelatinous macrozooplankton. The entire, unpreserved sample was analyzed right after catch using i) a light table, ii) a magnifying lamp with dark background or iii) a stereomicroscope. Sub-sampling was applied for abundant taxa. Size data were collected using conventional or electronic callipers.
Data source location	Collected in Northern Europe, extended North Sea area including the Skagerrak and Kattegat. Data stored at the National Institute of Aquatic Resources, Technical University of Denmark, DTU Aqua, Centre for Gelatinous Plankton Ecology and Evolution, 2800 Kgs. Lyngby, Denmark, and the Institute of Marine Research, Department of Aquatic Resources (SLU Aqua), Swedish University of Agricultural Sciences, 453 30 Lysekil, Sweden.
Data accessibility	Repository name: zenodo Data identification number: doi.org/10.5281/zenodo.13903034 Direct URL to data: https://zenodo.org/records/13903034 Appendix 1: Summary station information with/without size information Appendix 2: Raw dataset - gelatinous macrozooplankton, Q1-2022
Related research article	

## 1 Value of the Data

• The data is quantified during targeted ichthyoplankton and fisheries surveys (MIK-IBTS) and can provide unique insights into the coexistence of commercially important fish species and their gelatinous competitors and/or predators.
• This dataset is important for assessing the biodiversity and distribution of native and non-indigenous gelatinous macrozooplankton species in the North Sea and Skagerrak/Kattegat during winter (Q1 2022).
• The data is obtained through standardized protocols (North Sea - Midwater Ring Net (MIK) survey; [1,2]) which provides the possibility for close international collaboration.
• The data were generated during winter, which represents a time-point were plankton investigations are rare and only very few biodiversity assessments have been conducted to date.
• The data can help address the impact of rising winter temperatures and anthropogenic stressors on the biodiversity, distribution, and abundance patterns of gelatinous macrozooplankton.

## 2 Background

Data on gelatinous macrozooplankton diversity and distribution are sparse, especially during winter. The motivation for this dataset is to close this gap and engage with ichthyoplankton ecologists to quantify their bycatch during regular monitoring surveys such as the North Sea - Midwater Ring Net survey (MIK) [1]. The same methodology is applied during winter [3] and summer [5] surveys, hence methodologically consistent data can easily be attained.

## 3 Data Description

**Fig. 1:**
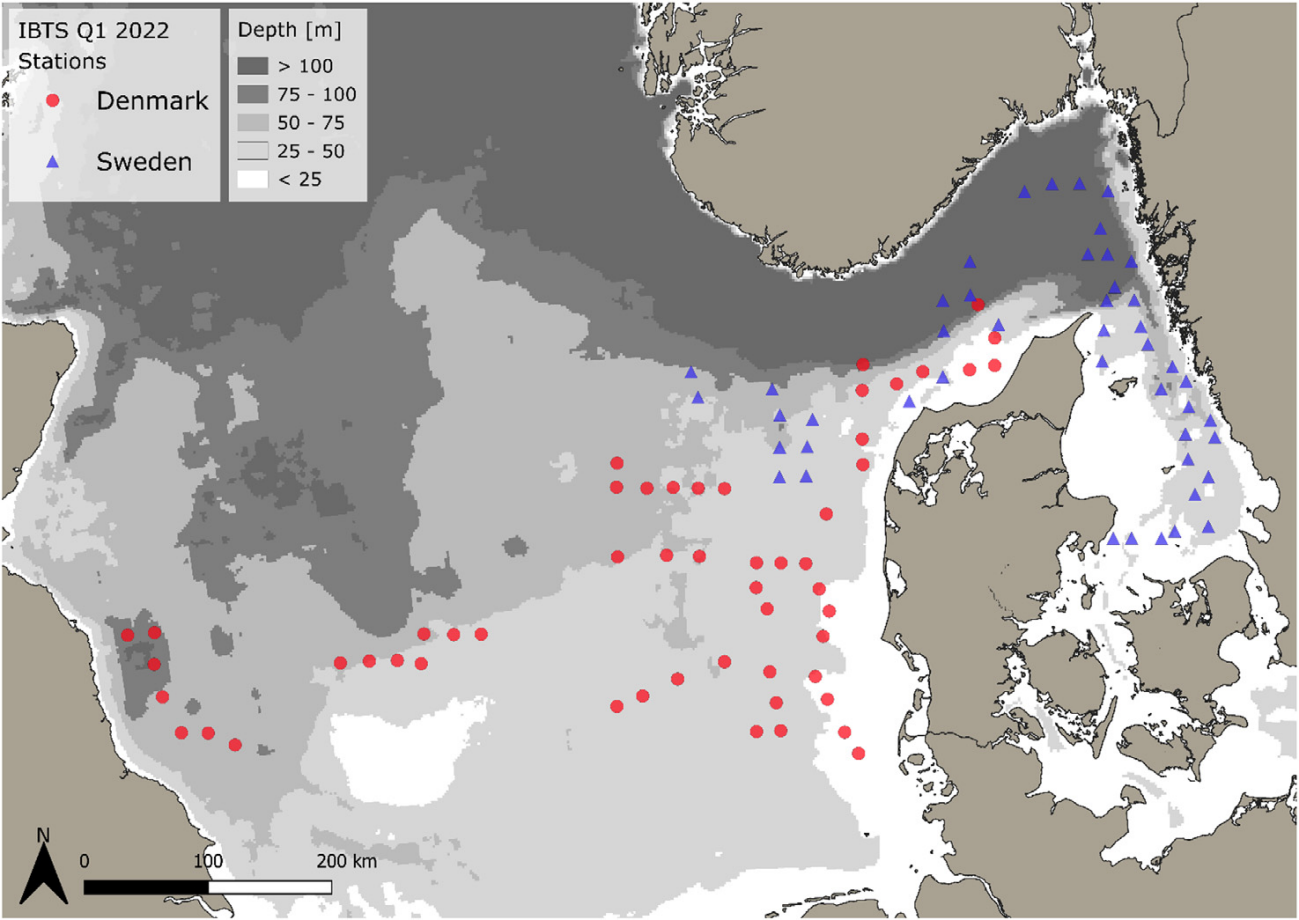
Gelatinous macrozooplankton sampling stations (*n* = 100) investigated during the International Midwater Ring Net (MIK) survey [1] as part of the International Bottom Trawl Survey (IBTS) from 20.1. to 12.2.2022. Samples from Danish (red circles) and Swedish (blue triangles) surveys are outlined covering the North Sea, Skagerrak and Kattegat in Northern Europe.

Spatial diversity distribution, species-specific abundance and biomass of the gelatinous macrozooplankton in the western, central and eastern part of the North Sea, Skagerrak and Kattegat [6] is presented in this data article. Data were collected as a part of the Danish and Swedish contribution to the North Sea - Midwater Ring Net (MIK) survey [1], an ichthyoplankton survey conducted at night-time during the quarter 1 (Q1) International Bottom Trawl Survey (IBTS) in 2022. One hundred stations were sampled from 20th of January to 12th of February 2022 (Fig. 1). The dataset consists of species-specific spatial distribution, abundance and size data. Further, the dataset includes estimated wet weights, based on published size-weight regressions, as originally reviewed and presented in Køhler et al. [3].

**Fig. 2:**
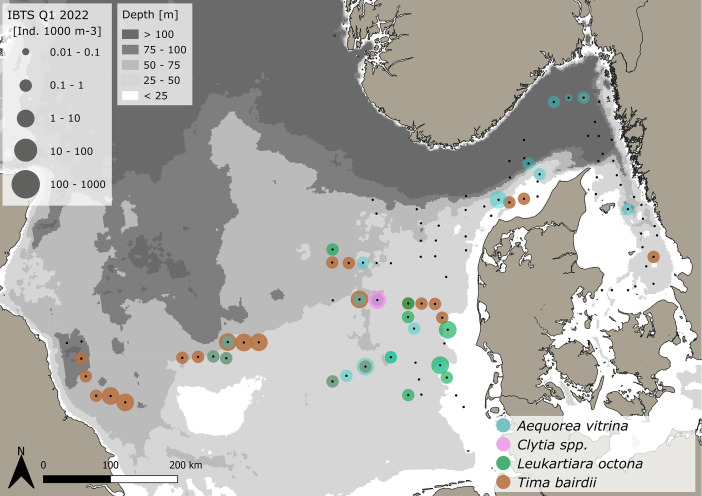
Distribution and abundance (individuals 1000 m^-3^) patterns of the hydrozoan species *Aequorea vitrina* (turkois)*, Clytia* spp*.* (rose)*, Leuckartiara octona* (green) *and Tima bairdii* (brown) for the North Sea and Skagerrak/Kattegat during January - February 2022. Black dots indicate sampling stations. Small hydrozoan species *Clytia* spp. and *L. octona* were not abundant and are therefore not quantitatively recorded in the Swedish dataset. (For interpretation of the references to color in this figure legend, the reader is referred to the web version of this article.)

**Fig. 3:**
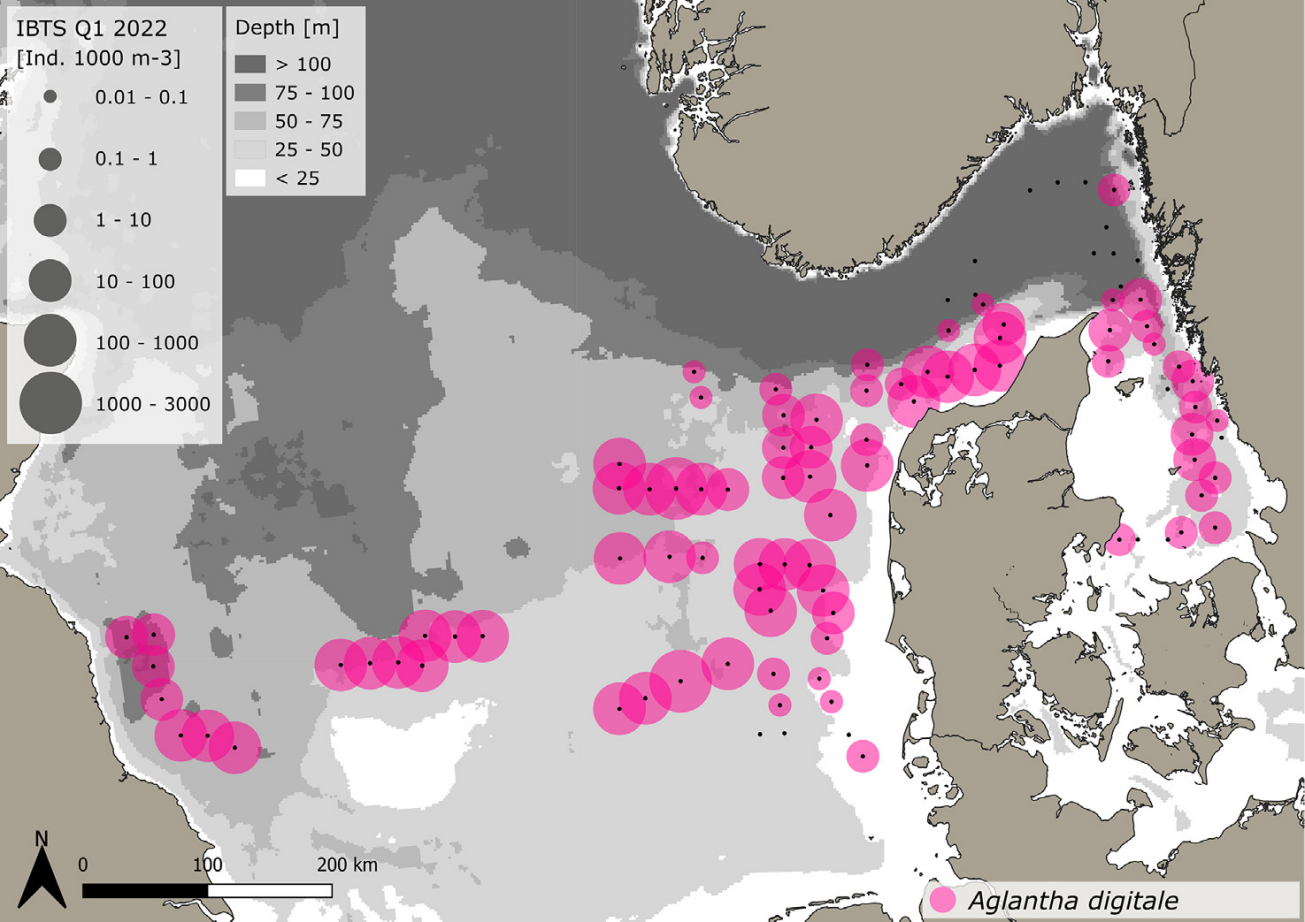
Distribution and abundance (individuals 1000 m^-3^) patterns of the hydrozoan *Aglantha digitale* across the North Sea and Skagerrak/Kattegat during January - February 2022. Black dots indicate sampling stations. Note: Swedish survey quantified *A. digitale* in abundance groups of +1 to +4 (see methods).

**Fig. 4:**
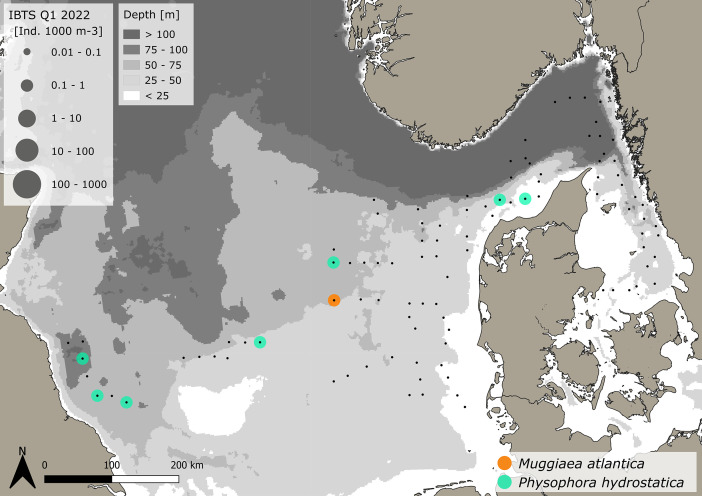
Distribution and abundance (1000 m^-3^) patterns of the hydrozoans *Muggiaea atlantica* (orange) and *Physophora hydrostatica* (green) in the North Sea and Skagerrak/Kattegat during January - February 2022. Black dots indicate sampling stations. (For interpretation of the references to color in this figure legend, the reader is referred to the web version of this article.)

**Fig. 5:**
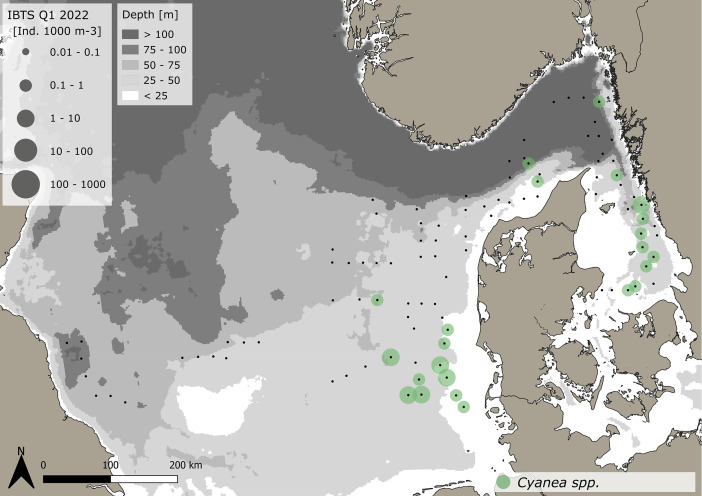
Distribution and abundance (individuals 1000 m^-3^) patterns of the scyphozoan jellyfish *Cyanea* spp. in the North Sea and Skagerrak/Kattegat during January - February 2022. Black dots indicate sampling stations.

**Fig. 6:**
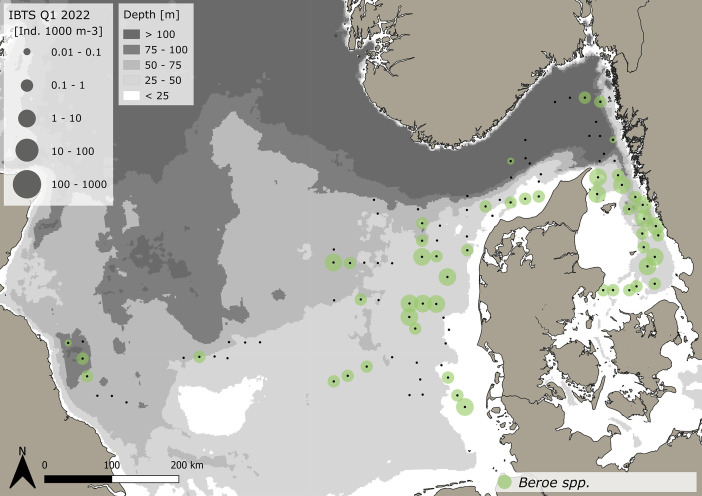
Distribution and abundance (individuals 1000 m^-3^) patterns of the comb jelly (ctenophora) *Beroe* spp. in the North Sea and Skagerrak/Kattegat during January and February 2022. Black dots indicate sampling stations.

**Fig. 7:**
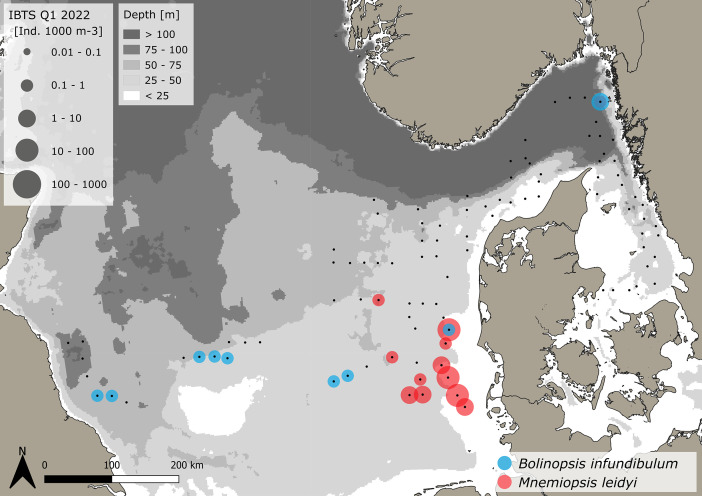
Distribution and abundance (individuals 1000 m^-3^) patterns of the native comb jelly (ctenophora) *Bolinopsis infundibulum* (blue) and the non-indigenous comb jelly *Mnemiopsis leidyi* (red) in the North Sea and Skagerrak/Kattegat during January and February 2022. Black dots indicate sampling stations. (For interpretation of the references to color in this figure legend, the reader is referred to the web version of this article.)

**Fig. 8:**
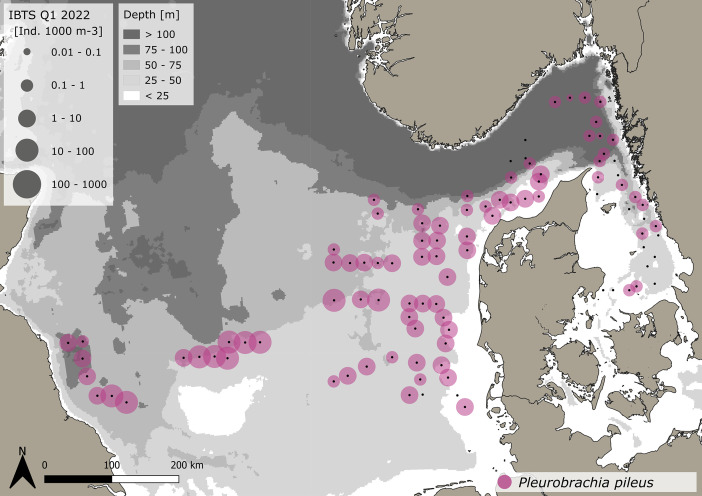
Distribution and abundance (individuals 1000 m^-3^) patterns of the native comb jelly (ctenophora) *Pleurobrachia pileus* (pink) in the North Sea and Skagerrak/Kattegat during January - February 2022. Black dots indicate sampling stations.

**Fig. 9:**
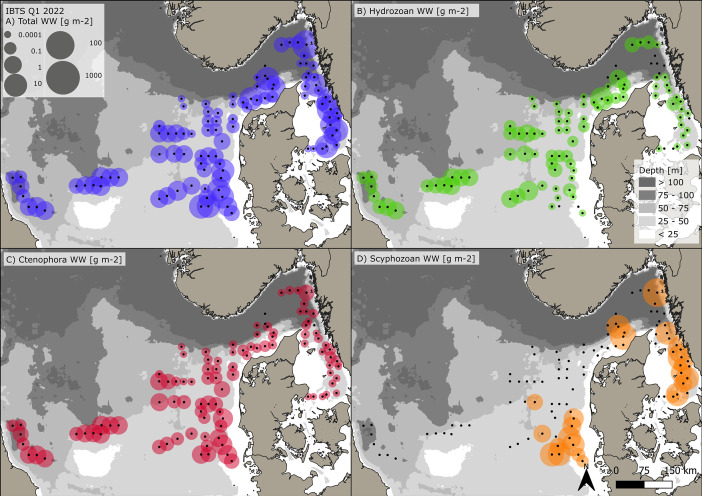
Gelatinous macrozooplankton biomass distribution (wet weight g m^-2^) across the North Sea and Skagerrak/Kattegat during January – February 2022 with A) total WW of all gelatinous macrozooplankton groups and split by groups with only, B) Hydrozoans, C) Ctenophora and D) Scyphozoans. Data originate from night-time ichthyoplankton work conducted during the Danish and Swedish Midwater Ring Net survey (MIK) as part of the International Bottom Trawl Survey (IBTS) Q1. Black dots indicate sampling stations.

The dataset consists of 71,888 geo-referenced, individual, species-specific gelatinous macrozooplankton abundance and biomass records. Species-specific abundance and distribution patterns for the groups i) hydrozoa, ii) scyphozoa and iii) ctenophora (Fig. 2, Fig. 3, Fig. 4, Fig. 5, Fig. 6, Fig. 7, Fig. 8) as well as wet weights for all groups and the total gelatinous macrozooplankton community (Fig. 9) are displayed. The dataset includes 13 gelatinous macrozooplankton species with seven hydrozoans: *Aequorea vitrina, Aglantha digitale, Clytia* spp.*, Leuckartiara octona, Tima bairdii, Muggiaea atlantica, Physophora hydrostatica;* two scyphozoans: *Cyanea lamarckii and Cyanea capillata* as well as four ctenophora: *Beroe* spp*., Bolinopsis infundibulum, Pleurobrachia pileus* and the non-indigenous *Mnemiopsis leidyi*. Due to the difficulty to separate early life stages (ephyra) of the scyphozoan jellyfish species *Cyanea capillata and C. lamarckii,* both species were grouped and are recorded as *Cyanea* spp*.* only. In the Swedish dataset, small and rare hydrozoans apart from the more abundant hydrozoans *A. digitale, T. bairdii and A. vitrina*, were not quantified. Note: in 2023, *Clytia* spp. was recorded in the Swedish dataset [4] and was also much more widespread and showed higher abundances in the Danish dataset, being recorded at 13 stations all along the Danish North Sea coast [4].

For the presented dataset, sizes were assessed for 4775 individuals and extrapolated to the entire dataset using average sizes of either the entire sample, sub-samples from that station or average sizes for the respective species from close by stations (see Tables 1, and 2 and methods for details). Sizes were subsequently used to estimate biomass by applying published length-weight (wet weight, WW) regressions, as reviewed and summarized in Køhler et al. [3]. Data are visualized (Fig. 2, Fig. 3, Fig. 4, Fig. 5, Fig. 6, Fig. 7, Fig. 8, Fig. 9) and a short data summary describing species-specific distribution characteristics across the sampled area is provided.

**Table 1:** Gelatinous macrozooplankton abundance and size characteristics for Danish Q1-2022 dataset. Total number of animals (n) for each species, standardized per volume (1000 m^-3^) and area (m^-2^) are provided as average (± SD) across all stations, as well as their respective maximum density. Sizes were estimated for all species from individual measurements or from sub-samples. For some stations, species-specific size information was missing and extrapolated (est. size) from nearby stations, as indicated by est. size / total number of stations this species was recorded at (total), as outlined in the last column. For example, *A. vitrina* was caught at 13 stations and size information was not available and estimated for 2 stations (2/13).

DK IBTS Q1 2022			Abundance		Abundance		Size			Stations
			(1000 m^-3^)		(m^-2^)		(mm)			
Class	Species	n	av. ± SD	max.	av. ± SD	max.	av. ± SD	min.	max.	est.size/total
Hydrozoa	*Aequorea vitrina*	38	0.6 ± 1.1	4.12	0.02 ± 0.03	0.11	135 ± 46.1	19	240	2/13
	*Aglantha digitale*	62,523	242 ± 393	2488	10.3 ± 17.1	102.5	7.9 ± 2	3	19	0/51
	*Clytia* spp.	42	9.89	9.89	0.43	0.43	6.3 ± 1.8	3	12	0/1
	*Leuckartiara octona*	19	0.5 ± 0.37	1.24	0.01 ± 0.005	0.02	7.3 ± 1.2	6	11	0/9
	*Muggiaea atlantica*	1	0.22	0.22	0.01	0.01	5.3	5	5	0/1
	*Physophora hydrostatica*	7	0.18 ± 0.05	0.23	0.008 ± 0.003	0.011	N/A	N/A	N/A	7⁎
	*Tima bairdii*	92	0.71 ± 0.52	1.84	0.04 ± 0.03	0.12	48.8 ± 10.2	20	87	0/22

Scyphozoa	*Cyanea* spp.	51	0.88 ± 0.78	2.6	0.03 ± 0.03	0.1	42.2 ± 35.6	10	150	0/13
Ctenophora	*Beroe* spp.	100	1.03 ± 1.23	4.76	0.04 ± 0.05	0.17	43 ± 16.2	9	187	10/23
	*B. infundibulum*	19	0.44 ± 0.18	0.67	0.02 ± 0.01	0.04	38.4 ± 8	28	47	6/8
	*Mnemiopsis leidyi*	503	11.5 ± 14.2	35.8	0.21 ± 0.23	0.64	24.1 ± 2.4	8	44	0/11
	*Pleurobrachia pileus*	2,671	8.88 ± 15.2	67.1	0.41 ± 0.71	3.34	16.7 ± 3.4	5	31	0/52

⁎ No size information.

**Table 2:** Gelatinous macrozooplankton abundance and size characteristics for the Swedish Quartal 1 - 2022 dataset. Total number of animals (n) for each species, standardized per volume (1000 m^-3^) and area (m^-2^) are provided as average (± SD) across all stations, as well as their respective maximum density. Sizes were estimated for all species from individual measurements or from sub-samples, apart from *A. digitale*, where average size was estimated from Danish survey stations as outlined by the extrapolated (est. size) to total station information provided in the last column - see Table 1 for specifics.

SW IBTS Q1 2022			Abundance		Abundance		Size			Stations
			(1000 m^-3^)		(m^-2^)		(mm)			
Class	Species	n	av. ± SD	max.	av. ± SD	max.	av. ± SD	min.	max.	est.size/total
Hydrozoa	*Aequorea vitrina*	7	0.13 ± 0.05	0.19	0.009 ± 0.004	0.014	103 ± 36.3	50	140	0/5
	*Aglantha digitale*	5,465	37.25 ± 49.1	151.3	1.1 ± 1.45	4.27	5	5	5	31/31
	*Tima bairdii*	3	0.18 ± 0.1	0.26	0.007 ± 0.002	0.009	35 ± 18	15	50	0/3
Scyphozoa	*Cyanea* spp.	16	0.46 ± 0.3	1.14	0.01 ± 0.01	0.04	60.8 ± 38.3	15	130	0/10
Ctenophora	*Beroe* spp.	128	1.05 ± 1.45	7.38	0.04 ± 0.055	0.27	17.7 ± 7.6	5	70	0/28
	*Bolinopsis infundibulum*	7	1.01	1.01	0.06	0.06	26⁎	15	30	0/1
	*Mnemiopsis leidyi*	1	0.14	0.14	0.01	0.01	25	25	25	0/1
	*Pleurobrachia pileus*	194	1.1 ± 1.7	6.72	0.04 ± 0.05	0.21	14.8 ± 5.1	5	35	0/31

⁎ Average size approximated.

***Aequorea vitrina* (Hydrozoa)** is present from the central North Sea to the Kattegat (Fig. 2). 45 individuals were caught at 18 stations, leading to an average abundances across the entire sampling region and dataset of 0.47 ± 0.95 *A. vitrina* 1000 m^-3^ (±SD) ranging from 0.06 to 4.12 *A. vitrina* 1000 m^-3^. Standardizing, taking depth differences between stations into account, led to an average area specific abundance of 0.02 ± 0.03 *A. vitrina* m^-2^ (±SD), with a range from 0.004 to 0.11 *A. vitrina* m^-2^. The average size (mm) for the entire dataset is 144 ± 46.9 (±SD) with a range of 19.4–240 mm. Average sizes for *A. vitrina* across stations were larger in the Danish compared to the Swedish investigation areas with 135 ± 46.1 mm to 103 ± 36.3 mm, respectively (see Table 1, Table 2).

***Clytia* spp. (Hydrozoa)** were caught at one station only during the Danish survey (Fig. 2) with a total of 42 individuals, leading to a density of 1 *Clytia* spp. 100m^-3^ or 0.43 *Clytia* spp. m^-2^ with an average size of 6.3 ± 1.8 mm (±SD), ranging from 3 to 12 mm (see Table 1).

***Leuckartiara octona* (Hydrozoa)** were caught at 9 stations in the Danish survey and were mainly distributed in the eastern North Sea (Fig. 2). A total of 19 individuals were caught with an average (±SD) and maximum density of 0.5 ± 0.37 and 1.24 *L. octona* 1000 m^-3^ or 0.01 ± 0.005 and 0.02 *L. octona* m^-2^. Sizes ranged between 6 and 11 mm, with an average (±SD) size across stations of 7.3 ± 1.2 mm (see Table 1).

***Tima bairdii* (Hydrozoa)** were caught at 25 stations throughout the sampling area, stretching from the western North Sea to the Kattegat. They were most abundant in the western-central North Sea (Fig. 2). A total of 95 animals were caught, leading to an average abundance (±SD) across the entire sampling region and dataset of 0.65 ± 0.52 *T. bairdii* 1000 m^-3^, ranging from 0.07 to 1.84 *T. bairdii* 1,000 m^-3^. The area specific abundance (±SD) across the entire dataset is 0.03 ± 0.03 *T. bairdii* m^-2^, ranging from 0.005 to 0.12 *T. bairdii* m^-2^. The average size (mm) across the entire datasets is 52.7 ± 13.3 mm (±SD), with a range from 15 to 87.2 mm (see Table 1, Table 2).

***Aglantha digitale* (Hydrozoa)** were caught at 82 stations throughout the sampling area stretching from the western North Sea to the Kattegat, but were most abundant in the central and eastern North Sea (Fig. 3). A total of 67,988 animals were caught, as estimated from samples, sub-samples and abundance groups. The average volume specific abundance across the entire dataset is 164.3 ± 326.1 *A. digitale* 1000 m^-3^, with a range between 0.23 and 2488.3 *A. digitale* 1000 m^-3^. Standardizing for depth, average area specific abundance across the entire dataset is 6.8 ± 14.2 *A. digitale* m^-2^, ranging between 0.004 and 102.5 *A. digitale* m^-2^. The average size across the entire dataset is 8.1 ± 2.3 mm (±SD), with a range between 3 and 18.9 mm. The specifics for the Danish and Swedish data are outlined in Table 1, Table 2, respectively.

***Muggiaea atlantica* (Hydrozoa - siphonophora)** were caught at 1 station in the central North Sea only during the Danish survey (Fig. 4) – see Table 1 for details.

***Physophora hydrostatica* (Hydrozoa - siphonophora)** were caught at 7 stations in the Danish sampling area stretching from the western North Sea to the Skagerrak (Fig. 4). The volume specific and area specific abundance as well as size range is provided in Table 1.

***Cyanea* spp. (Scyphozoa)** were caught at 23 station in the eastern North Sea and Skagerrak/Kattegat (Fig. 5). A total of 67 animals were caught. The average volume specific abundance across the entire dataset is 0.7 ± 0.64 *Cyanea* spp. 1000 m^-3^, with a range between 0.13 and 2.6 *Cyanea* spp. 1000 m^-3^. Standardizing for depth, average area specific abundance across the entire dataset is 0.002 ± 0.002 *Cyanea* spp. m^-2^, ranging between 0.003 to 0.1 *Cyanea* spp. m^-2^. The average size across the entire dataset is 40 ± 31.9 mm (±SD), with a range between 10 and 180 mm. The specifics for the Danish and Swedish data are outlined in Table 1, Table 2, respectively.

***Beroe* spp. (Ctenophora)** were caught at 51 stations throughout the sampling area stretching from the western North Sea to the Kattegat and were most abundant in the eastern North Sea and Skagerrak/ Kattegat (Fig. 6). A total of 228 animals were caught. The average volume specific abundance across the entire dataset is 1.04 ± 1.34 *Beroe* spp. 1000 m^-3^, with a range between 0.05 and 7.4 *Beroe* spp. 1000 m^-3^. Standardizing for depth, average area specific abundance across the entire dataset is 0.04 ± 0.05 *Beroe* spp. m^-2^, ranging between 0.003 and 0.27 *Beroe* spp. m^-2^. The average size across the entire dataset is 24.2 ± 21.2 mm (±SD), with a range between 5 and 187 mm. The specifics for the Danish and Swedish data are outlined in Table 1, Table 2, respectively.

***Bolinopsis infundibulum* (Ctenophora)** were caught at 9 stations, primarily located in the western-central North Sea with additional two stations in the Skagerrak and off the DK west coast (Fig. 7). A total of 26 animals were caught. The average volume specific abundance across the entire dataset is 0.5 ± 0.26 *B infundibulum* 1000 m^-3^, with a range between 0.22 and 1 *B infundibulum* 1000 m^-3^. Standardizing for depth differences, average area specific abundance across the entire dataset is 0.02 ± 0.02 *B infundibulum* m^-2^, ranging between 0.01 and 0.6 *B infundibulum* m^-2^. The average size across the entire dataset is 28.8 ± 8.6 mm (±SD), with a range between 15 and 47 mm. The specifics for the Danish and Swedish data are outlined in Table 1, Table 2, respectively.

***Mnemiopsis leidyi* (Ctenophora)** were caught at 12 stations primarily in the eastern North Sea (Fig. 7). A total of 504 animals were caught. The average volume specific abundance across the entire dataset is 10.6 ± 14 *M. leidyi* 1000 m^-3^, with a range between 0.14 and 35.8 *M. leidyi* 1000 m^-3^. Standardizing for depth, average area specific abundance across the entire dataset is 0.19 ± 0.23 *M. leidyi* m^-2^, ranging between 0.01 and 0.64 *M. leidyi* m^-2^. The average size across the entire dataset is 23.3 ± 5.6 mm (±SD), with a range between 7.8 and 44 mm. The specifics for the Danish and Swedish data are outlined in Table 1, Table 2, respectively.

***Pleurobrachia pileus* (Ctenophora)** were caught at 83 stations throughout the sampling area stretching from the western North Sea to the Kattegat, being most abundant in the western-central North Sea (Fig. 8). A total of 2865 animals were caught. The average volume specific abundance across the entire dataset is 5.97 ± 12.6 *P. pileus* 1000 m^-3^, with a range between 0.05 and 67.1 *P. pileus* 1000 m^-3^. Standardizing for depth, average area specific abundance across the entire dataset is 0.27 ± 0.59 *P. pileus* m^-2^, ranging between 0.005 and 3.34 *P. pileus* m^-2^. The average size across the entire dataset is 17.83 ± 3.7 mm (±SD), with a range between 4.6 and 35 mm. The specifics for the Danish and Swedish data are outlined in Table 1, Table 2, respectively.

## 4 Experimental Design, Materials and Methods

Gelatinous macrozooplankton was collected as a part of the North Sea - Midwater Ring Net survey (MIK) [1], an ichthyoplankton survey conducted at night-time during the quarter 1 (Q1) International Bottom Trawl Surveys (IBTS), on the Danish (R/V DANA, DTU Aqua, Denmark) and the Swedish (R/V SVEA, SLU, Sweden) research vessels, respectively. Sampling took place in the western, central, and eastern part of the North Sea (Danish sampling) and north-eastern North Sea, Skagerrak and Kattegat (Swedish sampling) from 20.1. to 12.2.2022. CTD casts were conducted to describe the physical environment in the sampling areas and are available through the International Council for the Exploration of the Seas (ICES) environmental database. Plankton sampling was conducted after sun set from approx. 18:00–06:00 (local time). The primary goal of night-time work is to catch herring larvae to provide a recruitment index for the stock assessment of the North Sea autumn spawning herring and further to assess the ichthyoplankton community in general. This procedure was extended and now includes gelatinous macrozooplankton assessment by Danish and Swedish partners [[3], [4], [5]]. Samples were collected from a total of 100 stations.

The methodology used on the Danish and Swedish surveys corresponds to the methodology of the North Sea Midwater Ring Net (MIK) survey [1,2] as previously described [3,5]. Gelatinous macrozooplankton was assessed from hauls with a MIK net, which is a large ring net with an opening diameter of 2 m and a 13 m long net bag with a mesh size of 1.6 mm apart from the last 1 m of the net bag and the cod end, which has a mesh size of 0.5 mm. The MIK net was deployed in double oblique hauls from the surface to 5 m above the bottom with a maximum depth of 100 m. The net included a calibrated flow meter in the center of the net opening and was towed at a ship speed of 3 knots. Further details about the MIK net and haul procedures can be found in the ICES MIK manual [2]. At the end of each haul, the net was carefully retrieved, washed, and the cod-end stored in a chiller with cold sea water until analysis in the ship based wet-laboratory. The entire cod-end content was analyzed for gelatinous macrozooplankton and fish larvae on a light table, a stereomicroscope (Danish data) or under a magnifying lamp with black background (Swedish data). During the Danish survey, all gelatinous zooplankton were identified to species or genera level, and further counted and measured to the nearest 0.1 mm with an electronic caliper connected to a laptop or a conventional caliper with a precision to the nearest 0.5 mm. During the Swedish survey, a conventional caliper was used for all gelatinous zooplankton organisms >0.5 cm plus the abundant hydrozoan species *A. digitale, T. bairdii* and *A. vitrina* (i.e. excluding other smaller and low abundant Hydrozoan species). Sub-sampling was conducted for very abundant taxa or when very high densities were observed at a station ensuring at least 20 individuals per sub-sample.

The water volume filtered during the tow was assessed by a calibrated flow-meter, which was located in the center of the net opening. The amount of filtered water in m^3^ was calculated as the Delta flow meter count (difference between end and start count), divided by the flow meter's calibration factor, multiplied with the net opening area [2]. Abundance per m^−3^ was estimated by dividing the total species count per net cast with the filtered water volume. The area specific abundance (individuals m^−2^) was estimated by the volume specific counts (individuals m^−3^) multiplied with the sampling depth (m) of the net. In the database and in the data presentation in this publication, volume specific counts are presented as individuals per 1000 m^−3^ (see Table 1, Table 2 for summary information, split by investigation country).

For the very abundant hydrozoan species *A. digitale*, the Swedish dataset consists of abundance groups and estimated densities, where >1–10 individual (presented by 1+), 11–100 individuals (2+), 101–1000 individuals (3+) and >1000 to 5000 (4+). Abundance estimates were set as an average abundance for each abundance group as 5, 50, 500 for the groups 1+ to 3+, respectively. Abundance group 4+ was not assigned. As size measurements for *A. digitale* were missing, we used an average size from surrounding Danish stations and assigned this average size to all Swedish stations in order to be able to calculate wet weights. For the Danish data, all *A. digitale* present in the sample were measured for 13 stations, while sizes were estimated from sub-samples with at least 20 individuals for the remaining 38 stations. Handling controls for sub-sampling have previously been conducted and showed a negligible impact on total abundance estimations - see Køhler et al. [3]. The spatial abundance and biomass distribution of gelatinous macrozooplankton and sampling stations (Fig. 1, Fig. 2, Fig. 3, Fig. 4, Fig. 5, Fig. 6, Fig. 7, Fig. 8, Fig. 9) were visualized using the freeware program QGIS 3.34.1 Prizen (https://www.qgis.org/en/site/index.html). Position information of sampled stations is provided in decimal form and plotted along with volume specific abundance (1000 m^−3^), area specific abundance (m^−2^) and area specific biomass (wet weight m^-2^). All data are available in the supplement and on Zenodo with the DOI: 10.5281/zenodo.13903034.

## Limitations

This data should be compared to other gelatinous macrozooplankton datasets generated by using ichthyoplankton MIK net surveys. Even though we quantitatively collected gelatinous macrozooplankton, the handling procedures introduce a bias and likely underestimates gelatinous macrozooplankton, especially siphonophore and ctenophore species. Irrespectively, if the same methodology is used over a long period, these data are extremely valuable and can inform about climate related responses of the gelatinous zooplankton community.

## Ethics Statement

The authors have read and follow the ethical requirements for publication in Data in Brief and confirming that the current work does not involve human subjects, animal experiments, or any data collected from social media platforms.

## CRediT Author Statement

**Camilla J. D. Jensen:** Data base compilation of national datasets with data quality control; data presentation including presentation of summary statistics; producing illustrations; writing of first manuscript draft and commenting on subsequent manuscript drafts. **Louise Køhler:** Data generation and responsible for Danish macrozooplankton sampling; laboratory work; data entry and initial Danish database compilation. **Bastian Huwer:** Conceptualization; responsible for Danish night-time sampling activities; supervision of macrozooplankton data generation; background data generation; commenting and editing final manuscript draft. **Malin Werner:** Data generation and responsible for Swedish macrozooplankton sampling; compilation of Swedish national dataset; quality control of Swedish national dataset and illustrations; commenting and editing final manuscript draft. **Leslie Cieters:** Initial draft of graphical illustrations. **Cornelia Jaspers:** Conceptualization; methodology; database compilation of national datasets with data quality control; re-analyses and supervision of data generation, data presentation and calculation of summary statistics; editing of illustrations; editing first draft and writing final manuscript draft. **All authors** read, commented and approved the final database and manuscript draft.

## Data Availability

ZENODOJensen et al. 2024 - Biodiversity and distribution of gelatinous macrozooplankton in the North Sea and adjacent waters dataset from winter 2022 - raw dataset (Original data). https://zenodo.org/records/13903034.

## Appendix Supplementary materials

Image, application 1

Image, application 2
